# Metabolic Role of Autophagy in the Pathogenesis and Development of NAFLD

**DOI:** 10.3390/metabo13010101

**Published:** 2023-01-07

**Authors:** Lingxuan An, Ulrich Wirth, Dominik Koch, Malte Schirren, Moritz Drefs, Dionysios Koliogiannis, Hanno Niess, Joachim Andrassy, Markus Guba, Alexandr V. Bazhin, Jens Werner, Florian Kühn

**Affiliations:** Department of General, Visceral, and Transplant Surgery, Ludwig-Maximilians-University Munich, 81377 Munich, Germany

**Keywords:** NAFLD, fibrosis, autophagy, metabolism

## Abstract

Non-alcoholic fatty liver disease (NAFLD) is a spectrum of liver disease, ranging from simple steatosis to hepatitis, fibrosis, cirrhosis, and hepatocellular carcinoma (HCC). Liver fibrosis, which portends a poor prognosis in NAFLD, is characterized by the excessive accumulation of extracellular matrix (ECM) proteins resulting from abnormal wound repair response and metabolic disorders. Various metabolic factors play crucial roles in the progression of NAFLD, including abnormal lipid, bile acid, and endotoxin metabolism, leading to chronic inflammation and hepatic stellate cell (HSC) activation. Autophagy is a conserved process within cells that removes unnecessary or dysfunctional components through a lysosome-dependent regulated mechanism. Accumulating evidence has shown the importance of autophagy in NAFLD and its close relation to NAFLD progression. Thus, regulation of autophagy appears to be beneficial in treating NAFLD and could become an important therapeutic target.

## 1. Introduction

NAFLD represents a spectrum of liver disease that can lead to progressive nonalcoholic steatohepatitis (NASH), fibrosis, cirrhosis, and hepatocellular carcinoma [1]. It is characterized by excessive fat accumulation in the liver that is not associated with high alcohol consumption [2]. Besides steatosis in hepatocytes, the lesion of NASH includes the presence of hepatocellular damage and inflammation. While simple steatosis is considered a benign disorder, NASH has become the second leading indication for end-stage liver disease and the need for liver transplantation, behind alcohol-related liver disease [3]. Obesity and type 2 diabetes mellitus are two major risk factors of NAFLD. As the epidemics of the two diseases increase worldwide, the prevalence of NAFLD increases proportionately [4]. NAFLD affects approximately one-quarter of the global adult population and carries a large economic burden [5]. Since no drugs have been approved by the FDA to treat NAFLD, it is urgently needed to find new therapeutic targets [6].

The liver is the central organ for fatty acid metabolism. Dietary intake of carbohydrates and lipids, fatty acids (FAs) derived from adipose tissue, can be used for gluconeogenesis, lipogenesis, and ketogenesis in the liver [7]. An imbalance in hepatic lipid homeostasis leads to metabolic disorders, resulting in fat accumulation within the liver. Different factors affecting hepatic lipid metabolism may promote fatty liver development. Autophagy is a cellular degradation and recycling process evolutionarily conserved in eukaryotes. The liver highly depends on autophagy as it mediates hepatocellular lipid metabolism. Thus, regulation of autophagy may serve as a therapeutic target in the treatment of NAFLD [8]. This review focuses on the metabolism-related effects of autophagy in different cells and their roles in the development of NAFLD.

## 2. Forms and Working Mechanisms of Autophagy

Autophagy was first described in the early 1960s, and it has already been proven that certain conditions, such as starvation, lead to its activation [9]. After feeding, autophagy components are suppressed by the nutrient-sensing mammalian target of rapamycin (mTOR) pathway. In mammals, three types of autophagy have been described: microautophagy, chaperone-mediated autophagy (CMA), and macroautophagy [10]. Microautophagy refers to the non-selective engulfment of cytoplasm by the lysosome [11]. It is coordinated with macroautophagy, CMA, and other self-eating pathways. For a long time, it was believed that microautophagy is more likely to occur in yeast than mammals since the yeast vacuole is much larger than the mammalian lysosome and can therefore engulf large LDs [12]. It was not until very recently that a study demonstrated that microlipophagy occurs and is not an insignificant mechanism for LD consumption [13]. There are still few studies about microlipophagy in the liver, and its role in NAFLD requires further research. CMA exclusively targets proteins and transfers them into the lysosomal lumen. In lipid metabolism, CMA targets perilipin 2 and 3 on the surface of LDs and promotes the recruitment of lipid lipases and macroautophagy machinery components.

Macroautophagy is the most prevalent form of autophagy and is considered the dominant activity for selectively trafficking LDs to the lysosome in hepatocytes. Macroautophagy (hereafter referred to as autophagy) involves the de novo formation of a phagophore to engulf cytoplasmic material to form double-membraned vesicles called autophagosomes. The subsequent fusion of autophagosomes with lysosomes forming autolysosomes degrades and recycles cargo to maintain cellular homeostasis [14].

Autophagy is regulated by a set of conserved genes called autophagy-related genes (ATGs). To date, more than 40 ATGs have been identified [15]. Autophagosome formation is induced by core ATG proteins. In *Saccharomyces cerevisiae* and *Magnaporthe oryzae*, autophagy is initiated in the pre-autophagosomal structure (PAS), which is localized in the vicinity of the vacuole [16]. In mammals, there may be multiple origins for phagophores, such as the endoplasmic reticulum (ER), outer membranes of the mitochondria, and the plasma membrane [17].

The autophagic process is initiated by nutrient starvation, followed by discontinuing TORC1 stimulation and subsequent activation of the Atg1 kinase complex [18]. The ULK1/2 complex, which is the mammalian ortholog of the yeast Atg1 complex, consists of ULK1/2, Atg13, focal adhesion kinase family-interacting protein of 200 kDa (FIP200), and Atg101 [19]. The kinase complex is one of the most upstream acting components of the autophagy machinery as it recruits downstream ATG proteins known as the Beclin1/ATG14/VPS15/VPS34 Class III PI3K complex to the autophagosome formation site and generates phosphatidylinositol 3-phosphate (PI3P) for the nucleation and formation of the autophagosomal phagophore [20].

The subsequent expansion and closure of the phagophore are mediated by two ubiquitin (Ubl)-like molecules, Atg12 and LC3/Atg8 [21]. Atg12 is conjugated to Atg5 and Atg16 to form the Atg12-Atg5-Atg16 complex. The Atg12-Atg5-Atg16 complex promotes the conjugation reaction of ubiquitin-like yeast Atg8 or mammalian LC3 proteins to lipid phosphatidylethanolamine (PE), resulting in a non-soluble form of Atg8-PE or LC3-II which presents on both the inner and outer sides of the autophagosomal membrane. The phagophore expands until it eventually engulfs intra-cellular cargo such as protein aggregates, organelles, and ribosomes in an autophagosome. In selective autophagy, cargo-bound autophagy receptors, for example, p62/SQSTM1, physically link their cargo to the autophagosomal membrane for eventual delivery to the lysosome [20,22,23,24].

In eukaryotic cells, autophagy occurs at a basal level and is responsible for the clearance of abnormal proteins, organelles, and infectious agents [25]. Dysfunction in autophagy has been implicated in various diseases, including cancer, neurodegenerative disease, and metabolic disorders [20,26,27,28].

## 3. Lipid Metabolism in the Liver

**Figure 1:**
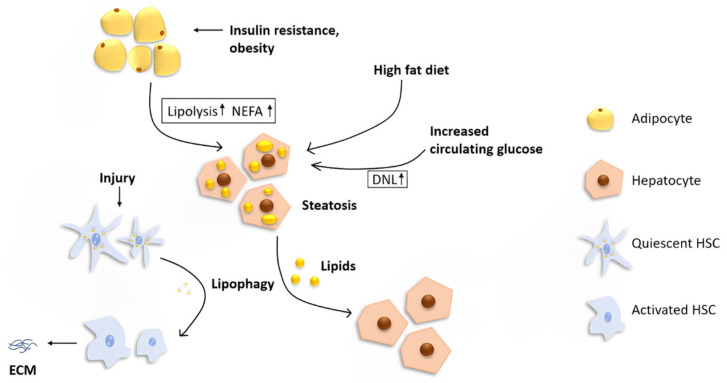
The role of lipophagy in the liver. Fat stored in the liver originates from three main sources: diet, DNL, and lipolysis from adipose tissues. A high-fat diet and increased circulating glucose cause increased lipid uptake and DNL, respectively. Insulin resistance and obesity lead to an increased level of lipolysis in adipocytes, resulting in a higher NEFA concentration in the systemic circulation. Increased uptake of lipids in hepatocytes leads to hepatic steatosis, which is the initial stage of NAFLD. Lipophagy plays different roles in different cells in the liver. In hepatocytes, lipophagy alleviates steatosis, while in quiescent HSCs, degradation of lipids via autophagy may fuel the cells for activation, resulting in increased extracellular matrix production and, ultimately, liver fibrosis.

The liver is the central organ that regulates lipid metabolism by fatty acid β-oxidation, lipogenesis, and lipoprotein uptake and secretion. However, the liver is not a storage depot for fat. Under normal conditions, the liver processes large quantities of fatty acids with less than 5% steady-state triglyceride (TG) storage [7]. Hepatocytes acquire lipids through diet, de novo lipogenesis (DNL), or uptake from circulation (Figure 1).

Dietary fat enters the liver as intestinally derived chylomicron remnants or spillover fatty acids [29]. These lipids reach systemic circulation through the intestinal lymphatic system and deliver triglycerides into muscle and adipose tissue for storage. During this process, chylomicron remnants are formed and taken up by the liver [7]. When carbohydrates are abundant, the liver converts non-lipid precursors, such as dietary sugars, into fatty acids, a process known as DNL. DNL of fatty acids takes place in the cytoplasm, where acetyl units derived from glucose or acetate are added to a precursor, acetyl-CoA, to synthesize fatty acid chains [30]. Acetyl-CoA undergoes subsequent condensation with a glycerol backbone. DNL is mediated by a series of coordinated enzymatic reactions, with the rate-limiting step in this pathway catalyzed by acetyl-CoA carboxylase that converts acetyl-CoA to malonyl-CoA. Increased circulating glucose levels upregulate lipogenic enzyme expression to encourage the storage of hepatic lipids, and DNL is abnormally increased in patients with NAFLD [29].

In the fasted state, most fatty acids enter the liver from lipolysis in adipose tissue (known as non-esterified fatty acid, NEFA) [7]. The liver takes NEFA from the blood in proportion to its concentration, and an influx of fatty acids quickly increases the hepatic lipid content [31,32]. The process of lipolysis in adipose tissue is highly hormone-related. Hormones regulate the lipolytic process by modulating lipolytic enzymes, for example, hormone-sensitive lipase (HSL) activity [33]. In the state of insulin resistance, insulin does not entirely suppress the activity of HSL in adipose tissue, leading to enhanced lipolysis and the release of fatty acids [34].

Excessive hepatic accumulation of NEFAs leads to cytotoxicity, resulting in cell injuries and cell death in hepatocytes [35]. To prevent lipotoxic damaged by excessive fatty acids’ oxidation and accumulation, hepatocytes convert the fatty acids into neutral lipids such as TG or cholesteryl and further deposit them into organelles called lipid droplets (LDs) [36]. Hepatocytes eliminate fatty acids by oxidation or very low-density lipoprotein (VLDL) secretion [31]. Under starvation, hepatocytes degrade LDs via lipolysis or autophagy [37]. Lipolysis is the metabolic process by which triacylglycerol (TAG) is broken down by hydrolysis into its constituent molecules: glycerol and free fatty acids (FFAs) [38]. The subsequent process of β-oxidation takes the FFAs and breaks them down into two-carbon acetyl-CoA molecules which enter the Krebs cycle to produce energy [7].

VLDL assembly is divided into two steps: the first involves the transfer of a small amount of lipid by the microsomal triglyceride transfer protein (MTP) to apolipoprotein; the second requires the fusion of apolipoprotein B-containing precursor particles with TG droplets to form mature VLDLs [39]. An elevated circulatory concentration is a major contributor to the development of atherosclerosis and cardiovascular disease, which is the leading cause of death in patients with NASH and type 2 diabetes [40,41].

Hepatic lipid homeostasis is supported by the coordinated regulation of lipid uptake, de novo synthesis, and elimination. The overflow of any metabolite pool can lead to organ dysfunction and subsequent pathologies.

## 4. The Role of Autophagy and Its Regulation in Lipid Metabolism within the Liver

The regulatory effect of autophagy on lipid metabolism was first demonstrated by Singh et al. [27]. The study showed that autophagy contributes to the degradation of LDs in hepatocytes. Since then, many studies have unraveled the role of autophagy in lipid metabolism in vivo and in vitro. Since the naming of specific autophagy pathways depends on the substrate that is degraded, the highly selective form of LD-targeted autophagy is known as “lipophagy”.

In the liver, two major pathways mediate the breakdown of TAGs stored in LDs for subsequent oxidation: lipolysis and lipophagy [42]. Lipolysis involves cytosolic lipases, including adipose triglyceride lipase (ATGL), HSL, and monoglyceride lipase (MGL), acting in sequence to catalyze the release of the three fatty acid moieties comprising the TAG molecule, with ATGL acting as the rate-limiting lipase [43]. The FFAs released by this process can provide substrates for mitochondrial β-oxidation or serve as potent signaling molecules for various cellular processes; at the same time, these FAs can be re-esterified back into TAG for storage [44].

Lipophagy involves the formation of autophagosomes, which pinch part of the LD and then fuse with lysosomes to provide the liver with FFAs that can be used as an energy source [45]. Though the relative utilization of lipolysis versus lipophagy is presently unclear, it is believed that the two process operate in tandem, as a previous study showed that inhibition of autophagy does not have an additive effect on lipolysis inhibition-mediated lipid accumulation [27]. Another study observing the size of LDs showed that the inhibition of ATGL resulted in about four times larger cytoplasmic LDs than lysosomal inhibition did [44]. Moreover, ATGL inhibition affected LD size at earlier time points than lysosomal acid lipase (LAL) inhibition did, suggesting that lipolysis targets these LDs upstream of lipophagy. Dysfunction of autophagy leads to excessive lipid accumulation in hepatocytes, resulting in fatty liver disease.

Various factors can regulate the process of lipophagy through different pathways. Understanding these pathways may provide an attractive approach to preventing NAFLD. As autophagy is usually suppressed by amino acids and insulin through the mTOR- or/and Akt-dependent pathways, suppressed lipophagy is observed in the liver of mice with insulin resistance and hyperinsulinemia induced by a high-fat diet (HFD) [46,47]. On the contrary, hormones that regulate catabolic processes, for example, adrenaline and thyroid hormone, exhibit autophagy-promoting effects [48,49,50]. A fasting-induced hormone, fibroblast growth factor 21 (FGF21), has been shown to promote lipophagy. Previous studies showed that FGF21 deficiency impairs hepatic lysosomal function by blocking Transcription factor EB (TFEB), a master regulator of lysosome biogenesis and autophagy [51,52].

Lipophagy is also regulated by transcriptional regulators. TFEB, a basic helix-loop-helix leucine zipper protein, is the most studied transcriptional regulator of lipophagy [53]. TFEB regulates genes involved in several steps of lipid catabolism, and it regulates autophagic flux by promoting the biogenesis of lysosomes, the formation of autophagosomes, and fusion with lysosomes. Another transcription factor, TFE3, which is also a member of the basic helix-loop-helix leucine zipper family of transcription factors, can bind to the coordinated lysosomal expression and regulation (CLEAR) element and regulate autophagy flux and lysosome function. Overexpression of TFE3 was shown to alleviate hepatocellular steatosis markedly [54].

The nuclear receptor PPARα belongs to the peroxisome proliferator-activated receptors (PPARs) family, which plays an essential role in lipid metabolism. PPARα is highly expressed in the liver, and its activation lowers lipid levels [55]. Pharmacological activation of PPARα reverses normal inhibition of autophagy in the fed state [56]. Interestingly, PPARα and the bile acid receptor farnesoid X receptor (FXR) compete for binding to shared sites in autophagic gene promoters. It was shown that FXR strongly suppresses lipophagy in the fasting state.

FXR is activated by increased bile acid levels after feeding. As bile acids regulate postprandial hepatic transition from a catabolic to an anabolic state, they inhibit hepatic autophagic activity [57,58]. A previous study showed that activation of FXR inhibited autophagy, which is independent of mTOR activation. Further, ChIP-seq detected the top scoring motifs for cAMP response element-binding protein (CREB), another transcriptional activator that promotes lipophagy during fasting, in FXR binding peak regions [59]. The study identified an FXR–CREB axis as the key regulator of autophagy during feeding/fasting cycles.

## 5. Autophagy and Hepatic Stellate Cell Activation

While lipophagy appears beneficial in attenuating steatosis in hepatocytes, lipophagy in HSCs is thought to exacerbate its activation and lead to liver fibrosis (Figure 1). Liver fibrosis is a repair process of chronic injury with excessive accumulation of extracellular matrix [60]. The activation of HSCs after liver injury is the principal event underlying hepatic fibrogenesis [61]. HSCs are resident non-parenchymal cell types and are normally filled with LDs containing retinyl esters and triglycerides. Upon liver injury, quiescent HSCs are activated and converted into myofibroblasts, which release a large amount of ECM [62,63]. Stellate cell activation is likely to be an intensely energy-demanding process to promote pathways for cell proliferation, extracellular matrix secretion, and cell contraction [64]. A significant feature of HSC activation is the release of LDs containing retinyl esters and triglyceride. At the same time, they acquire myofibroblast-like features, such as the expression of smooth muscle alpha actin and de novo expression of receptors for fibrogenic, chemotactic, and mitogenic factors [65]. Autophagy acts on LD release and can thus provide energy for HSC activation.

Inhibition of autophagy in HSCs has shown the effect of fibrosis attenuation in vivo and in vitro [64]. Consistent with attenuated HSC activation, autophagy inhibition leads to preserved LDs in HSCs and a decrease in total ATP levels [62]. Since inflammation is important for HSC activation, the gut-derived Gram-negative bacterial cell wall component lipopolysaccharide (LPS), a molecule that strongly stimulates inflammation, has long been demonstrated to be associated with HSC activation [66]. The study by Chen et al. [67] showed the regulatory effect of LPS in HSC activation through autophagy. LPS treatment promoted lipophagy in HSCs, and this effect was involved in the LPS-mediated reduction in Bambi expression. Bambi, on the other hand, is the transforming growth factor-β (TGFB) pseudoreceptor. A decrease in the expression of Bambi can sensitize HSCs to TGFB activation, which leads to liver fibrosis. As NAFLD patients commonly show an impaired gut barrier function, LPS derived from the gut lumen enters circulation [68]. Blood LPS levels have been shown to be higher in patients with NAFLD [69]; LPS-mediated liver injury through autophagy induction in HSCs may be an essential mechanism underlying liver fibrosis development.

In recent years, a microarray analysis of hepatic miRNAs identified 19 upregulated and 18 downregulated miRNAs in mouse livers with fibrosis [70]. One of the downregulated miRNAs in activated HSCs, miR-30a, showed the effect of ameliorating hepatic fibrosis by inhibiting Beclin1-mediated autophagy. Increased LDs in HSCs treated with miR-30a were also observed, indicating that miR-30a, lipophagy, and hepatic fibrosis are related [71]. Zhang et al. [72] demonstrated the important role of reactive oxygen species (ROS) in autophagy during HSC activation. The study showed that cellular ROS gradually accumulate during HSC activation; this process also facilitates autophagy activation in HSCs. ROS scavenger treatment abrogated ROS and resulted in impaired autophagy and LD disappearance. Meng et al. [73] showed that carvedilol, a recommended drug for treating portal hypertension, can alleviate liver fibrosis by inhibiting autophagic flux and subsequently inducing apoptosis in HSCs.

However, those results are controversial since some of the studies demonstrated the beneficial role of autophagy in liver fibrosis. For example, the study by Zhang et al. [74] showed that the anti-fibrosis effect of methyl helicterate might depend on its apoptosis and autophagy-inducing mechanism. Further experiments showed that inhibiting autophagy abolished this effect. A similar effect of autophagy of enhancing caffeine-induced apoptosis in HSCs was observed [75]. Interestingly, none of the studies supporting the induction of autophagy to alleviate liver fibrosis are related to lipophagy.

## 6. Autophagy and Gut Barrier Function

The gut and the liver are anatomically and physiologically connected, and their relationship is called the “gut–liver axis” [76]. Previous studies demonstrated the role of gut function in liver diseases, as dysfunction of the gut barrier leads to more toxic substances traveling to the liver through the portal vein, which leads to chronic inflammation in hepatocytes and liver fibrosis [77,78,79,80]. Maintaining gut homeostasis is crucial in preventing NAFLD, as the intestinal epithelium forms a physical barrier against toxins and pathogenic organisms.

**Figure 2:**
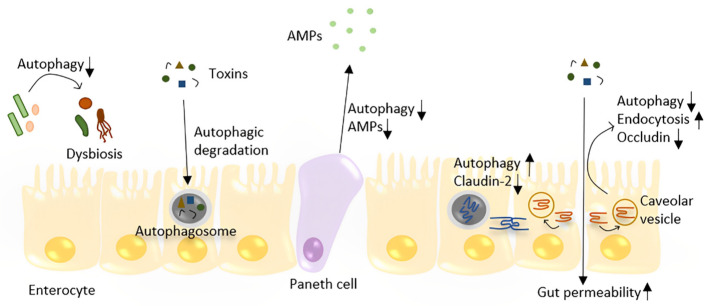
The role of autophagy in the maintenance of gut homeostasis. Autophagy plays a role in the defense against toxins and bacteria by directly degrading them in enterocytes. Autophagy regulates the TJ barrier through two different pathways: by degrading pore-forming claudin-2 and preventing occludin endocytosis, which prevents increased gut permeability. Depleting autophagy in the gut leads to dysbiosis. In Paneth cells, decreased autophagy may lead to less AMP production and Paneth cell apoptosis.

Pioneering studies showed mutations in autophagy-associated genes as susceptibility factors for Crohn’s disease [81]. Subsequent studies showed that autophagy is pivotal for intestinal homeostasis maintenance. Autophagy regulates gut homeostasis mainly through four mechanisms: pathogen degradation, inhibition of intestinal cell apoptosis, enhancing tight junction proteins, and avoiding gut microbiome dysbiosis (Figure 2).

Intestinal epithelial autophagy is an essential mechanism for eliminating invading bacteria. A previous study showed that autophagosomes form within 24 h in the small intestinal epithelium after oral *S. typhimurine* infection in mice [82]. Immunofluorescence staining showed the signal of colocalization of autophagosomes and intracellular bacteria. Moreover, intestinal epithelial cell-specific Atg5 gene-deficient mice showed an increased amount of *S. typhimurine* both in intestinal epithelial cells and extraintestinal tissues, indicating that autophagy plays a role in limiting bacterial dissemination. A similar study described the role of the autophagy-related gene Atg16l1 in bacteria clearance [83]. In addition, epithelial Atg16l1 deficiency leads to fewer Paneth cells and less antimicrobial peptide (AMP) production in mice, indicating the role of Atg16l1 in antibacterial defense. Atg16l1 also showed a cytoprotective function for intestinal epithelial cells in mouse models and organoids, and this effect is related to mitochondrial homeostasis as Atg16l1-deficient intestinal organoids showed increased ROS accumulation [84]. Another study showed that mice lacking Atg14 within the intestinal epithelium developed widespread small intestinal villus atrophy. In vitro experiments showed that Atg14 protects intestinal epithelial cells from tumor necrosis factor (TNF)-mediated programmed cell death [85]. Intestinal stem cells which reside at the bottom of the intestinal crypts are crucial for epithelial repair. Studies have shown the effect of Atg5 and Atg7 in maintaining intestinal stem cell integrity [86,87], indicating that autophagy is necessary for intestinal regeneration.

Intracellular tight junctions (TJs) are essential to the gut barrier; they function as gatekeepers for the paracellular pathway. Disruption of the intestinal TJ barrier leads to the permeation of pro-inflammatory molecules from the gut lumen into the circulating system, which is considered a risk factor for NAFLD development [88]. Autophagy strengthens the intestinal TJ barrier through two distinct mechanisms: by targeting pore-forming claudin-2 protein degradation and/or inhibiting the degradation of occludin by preventing its caveolar endocytosis from the membrane [89,90]. Autophagy also protects against LPS- and TNFα-induced intestinal injury in vivo as rapamycin significantly attenuated LPS- and TNFα-induced increases in intestinal permeability [90]. Emerging evidence supports that the gut microbiota may contribute to liver diseases through multiple mechanisms influenced by bacterial composition [91]. The reciprocal interactions between the gut microbiota and autophagy have been well described in previous reviews [92,93]. In general, autophagy deficiency leads to an altered composition and diversity of the gut microbiota, resulting in dysbiosis. Gene function enrichment analysis of the gut microbiota showed enriched pathways of infectious disease in small intestinal segments of Atg5^−/−^ mice, indicating that autophagy-deficient mice may be susceptible to pathogenic bacteria [94]. A recent study observed the role of estrogen-related receptor alpha (ESRRA), an autophagy regulator, in maintaining intestinal homeostasis. Depleting ESRRA led to the inhibition of autophagic flux and dysbiosis [95]. The above studies show that restoring intestinal autophagy is of great significance for maintaining the intestinal barrier and may be used as a treatment to prevent the deterioration of NAFLD.

## 7. Potential Therapeutics and Future Directions

The current first-line treatment of NAFLD is lifestyle intervention targeting weight loss through exercise and a hypocaloric diet [96]. Bariatric surgery has shown the effect of ameliorating comorbidities and improving mortality from malignancy and cardiovascular disease in most patients with NAFLD. Though bariatric surgery is not recommended as a specific treatment for NAFLD, it is the best option for weight reduction if lifestyle modifications and pharmacological therapy have not yielded long-term success [97,98].

Although autophagy is an old concept, its research in liver disease is still at a relatively early stage. In recent years, the role of autophagy in NAFLD has attracted the attention of the academic community and has been extensively studied [99]. Autophagy is complex in NAFLD as it plays different roles within specific tissues and cells and varies during different stages of disease. The potential of autophagy in the treatment of NAFLD is huge because the role of autophagy in each organ can be used as a therapeutic target. Moreover, some existing treatments have been proven to be related to the regulation of autophagy and shown potential treatment effects. For example, metformin, one of the most used first-line drugs for type 2 diabetes mellitus, was shown to induce autophagy in hepatocytes and improve liver function in NAFLD patients [100,101,102]. Since mTORC1 is an inhibitor of autophagy, inhibiting mTORC1 by deleting folliculin protein protected mice from developing NAFLD [103,104]. A steatosis-attenuating effect was also observed in rapamycin-treated mice [105]. Thus, inhibiting mTORC1 is a promising strategy for treating NAFLD [106]. Some new strategies, such as fecal microbiota transplantation (FMT), which can promote gut barrier integrity and treat liver diseases, have been demonstrated to beneficially regulate autophagy in intestinal mucosa [107], indicating that regulating autophagy is feasible and realistic for the treatment of NAFLD. Another approach for the treatment of liver fibrosis in mice, intestinal alkaline phosphatase (IAP) [108], induces autophagy in HCT116 epithelial cells [109]. Whether it has an autophagy-related role in reducing liver fibrosis needs further verification.

Calorie restriction is the most robust modifiable inducer of autophagy and is a natural dietary therapy that improves health and extends longevity [110]. Autophagy has been an emerging target for cancer therapy, and intermittent fasting (IF) has been shown to improve the chemotherapeutic response [111]. Current evidence suggests IF in patients with NAFLD is a feasible, safe, and effective means for weight loss [112]. Since most studies use non-invasive testing (NIT), it is difficult to demonstrate the relationship between fasting and improvement in NAFLD histology. Moreover, the long-term feasibility and safety of IF should be further studied.

## 8. Conclusions

Even though current data on the therapeutic effects and mechanism of autophagy in liver diseases are mostly based on in vitro and animal experiments, there is accumulating evidence that abnormal autophagy in the liver is closely related to NAFLD progression. Thus, the regulation of autophagy appears to be beneficial in treating NAFLD and could become an important therapeutic target.
